# Pre- and Intraoperative Visualization of GRPR-Expressing Solid Tumors: Preclinical Profiling of Novel Dual-Modality Probes for Nuclear and Fluorescence Imaging

**DOI:** 10.3390/cancers15072161

**Published:** 2023-04-05

**Authors:** Marjolein Verhoeven, Maryana Handula, Lilian van den Brink, Corrina M. A. de Ridder, Debra C. Stuurman, Yann Seimbille, Simone U. Dalm

**Affiliations:** 1 Department of Radiology and Nuclear Medicine, Erasmus Medical Center, University Medical Center Rotterdam, 3015 GD Rotterdam, The Netherlands; 2 Department of Experimental Urology, Erasmus Medical Center, University Medical Center Rotterdam, 3015 GD Rotterdam, The Netherlands; 3 Life Sciences Division, TRIUMF, Vancouver, BC V6T 2A3, Canada

**Keywords:** gastrin-releasing peptide receptor (GRPR), image-guided surgery, dual-modality probe, NeoB, optical imaging, SPECT imaging, multi-modality imaging

## Abstract

**Simple Summary:**

Image-guided surgery is a technique that can help the surgeon detect and remove tumors more precisely. Using a tumor-targeting agent with both a radioactive and a fluorescent label allows us to combine the benefits of two imaging modalities; preoperative nuclear imaging for tumor localization and intraoperative fluorescence imaging for precise and real-time visualization of tumor tissue during surgery. The gastrin-releasing peptide receptor (GRPR) is a promising target for this application because of its overexpression in several solid tumors, e.g., prostate and breast cancers. In this study, a full preclinical characterization of four previously developed GRPR-targeting dual-modality probes is presented, including the characterization of the biodistribution profile, selection of the optimal probe and a proof-of-concept for image-guided surgery. This project is the first comprehensive investigation of the effect of linker modifications and administered dose on the in vivo behavior of GRPR-targeting dual-modality probes, and provides a basis for clinical translation.

**Abstract:**

Image-guided surgery using a gastrin-releasing peptide receptor (GRPR)-targeting dual-modality probe could improve the accuracy of the resection of various solid tumors. The aim of this study was to further characterize our four previously developed GRPR-targeting dual-modality probes that vary in linker structures and were labeled with indium-111 and sulfo-cyanine 5. Cell uptake studies with GRPR-positive PC-3 cells and GRPR-negative NCI-H69 cells confirmed receptor specificity. Imaging and biodistribution studies at 4 and 24 h with 20 MBq/1 nmol [^111^In]In-**12**-**15** were performed in nude mice bearing a PC-3 and NCI-H69 xenograft, and showed that the probe with only a *p*ADA linker in the backbone had the highest tumor-to-organ ratios (T/O) at 24 h after injection (T/O > 5 for, e.g., prostate, muscle and blood). For this probe, a dose optimization study with three doses (0.75, 1.25 and 1.75 nmol; 20 MBq) revealed that the maximum image contrast was achieved with the lowest dose. Subsequently, the probe was successfully used for tumor excision in a simulated image-guided surgery setting. Moreover, it demonstrated binding to tissue sections of human prostate, breast and gastro-intestinal stromal tumors. In summary, our findings demonstrate that the developed dual-modality probe has the potential to aid in the complete surgical removal of GRPR-positive tumors.

## 1. Introduction

Among cancer types, solid cancers have the highest incidence, with breast, lung and prostate cancer accounting for nearly a third of all newly diagnosed cases in 2020 [1]. Surgery is the most common treatment for these cancers, and complete tumor resection is fundamental to its success. However, one of the greatest challenges for surgeons is to identify tumor boundaries intraoperatively. For prostate cancer, for example, the percentage of resected specimens with cancer cells present at the resection border is, on average, as high as 21.03% [2]. These positive surgical margins have been associated with an increased risk of tumor recurrence and a consequent increased administration of adjuvant treatments [3,4,5]. Hence, there is an important need to improve the accuracy of tumor resection.

One strategy that has been proposed to aid tumor identification and delineation is image-guided surgery [6]. A breakthrough in this field is the recent FDA approval of the first tumor-targeting agent for fluorescence-guided surgery, commercially called CYTALUX. The intraoperative use of CYTALUX revealed additional lesions in 27% of ovarian cancer patients that would otherwise have been missed, clearly illustrating the benefit of image-guided surgery [7]. However, fluorescence imaging is unfortunately limited by penetration depth, and, therefore, the combined use of nuclear and optical imaging modalities has been proposed as a strategy to further improve surgical navigation. This dual-modality concept is based on both preoperative and intraoperative illumination of cancerous tissue. Nuclear imaging has proved to be a highly sensitive technique for preoperatively detecting cancer, and the radioisotope can additionally be used to locate deeper lesions intraoperatively [8]. Complementary to this, intraoperative optical imaging can improve the surgical view of tumor boundaries in real time. Accordingly, dual-modality nuclear/fluorescent imaging probes would potentially possess the excellent sensitivity and high penetration depth of nuclear imaging and the high, but shallow, resolution and real-time visualization capability of fluorescence imaging in a single agent [9].

The gastrin-releasing peptide receptor (GRPR) is considered an attractive target for molecular imaging, as it is overexpressed in several high-incidence solid tumors, such as prostate, breast, lung and colon cancer [10]. For this reason, multiple GRPR-targeting radiopharmaceuticals and fluorescent agents have been developed and successfully evaluated in preclinical and/or initial clinical studies [11,12,13,14,15]. Among them is the potent DOTA-coupled peptide NeoB (formerly called NeoBOMB1), which has shown good tumor-targeting ability and favorable pharmacokinetics in vivo [16,17]. Due to these promising characteristics, we functionalized NeoB with the sulfo-cyanine 5 (sCy5) far-red fluorescent dye to facilitate its bimodal use. In our previously published work, we described the synthesis of four novel dual-modality probes based on the NeoB backbone and with different linker structures. These probes presented with a good GRPR binding affinity in vitro, and for two of the dual-modality probes, we showed promising tumor uptake in a pilot preclinical biodistribution and imaging study [18].

In the present study, we further characterized the four developed GRPR-targeting dual-modality probes. First, we determined the biodistribution profile of all four probes at two time points and selected the dual-modality probe and time point that resulted in optimal tumor-to-background ratios (TBR). Second, the relationship between dose and TBR was investigated for both imaging techniques to achieve maximal imaging contrast. Lastly, we mimicked image-guided tumor resection in a small proof-of-concept study and performed an ex vivo binding study on a wide range of solid cancers to illustrate the translational utility of our selected dual-modality probe. Ultimately, we aim to improve surgical efficacy for patients with GRPR-expressing solid tumors and thereby reduce the likelihood of tumor recurrence.

## 2. Methods

### 2.1. Chemistry and Radiolabeling

**Figure 1:**
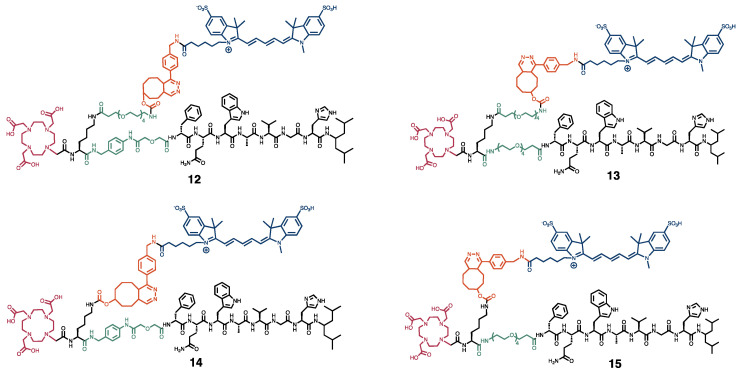
Chemical structures of the four dual-modality probes **12**-**15**. In black, the peptide sequence responsible for binding to the GRPR, and the lysine residue. In green, the PEG_4_ and *p*ADA linkers. In red, the DOTA chelator. In orange, the inverse electron demand Diels–Alder click reaction, and in blue, the sulfo-cyanine 5 fluorescent dye.

The synthesis and radiolabeling of the dual-modality probes was reported previously [18]. A total of four probes were generated by introducing a linker (p-aminomethylanilinediglycolic acid (*p*ADA) or PEG_4_) and lysine between the binding domain of NeoB and DOTA chelator, in combination with or without a PEG_4_ linker coupling the lysine and trans-cyclooctene (TCO) moiety (Figure 1). A short overview of the methodology can be found in the Supplementary Materials.

The radiolabeling of the four dual-modality probes with [^111^In]InCl_3_ (Curium, Petten, The Netherlands) was performed in water containing Kolliphor^®^ HS 15 (Merck, Amsterdam, The Netherlands) (0.5 mg/mL) in the presence of sodium acetate, gentisic and ascorbic acid, which acted as radiolysis quenchers [19]. The radiolabeling mixture was incubated at 90 °C for 20 min. Then, diethylenetriaminepentaacetic acid was added to complex free radionuclide. The radiochemical yield (>95%) and radiochemical purity (>95%) of the radiopeptides were determined using instant thin-layer chromatography and radio high-performance liquid chromatography, respectively. The radiolabeled dual-modality probes were prepared with a molar activity of 20 MBq/nmol for all in vitro experiments.

### 2.2. Cell Culture

The GRPR-positive human-derived prostate adenocarcinoma epithelial PC-3 cells (ATCC, Manassas, VA, USA) and the GRPR-negative human small-cell lung carcinoma NCI-H69 cells (ECACC, Salisbury, UK) were cultured in F-12K Nutrient mix (Gibco, Paisley, UK) and RPMI medium 1640 + GlutaMAX-I (Gibco), respectively. Both media were supplemented with 10% fetal bovine serum (FBS; Gibco), penicillin (100 units/mL; Sigma-Aldrich, Darmstadt, Germany) and streptomycin (100 µg/mL; Sigma-Aldrich). Cell lines were routinely passaged and grown at 37 °C in a humidified atmosphere with 5% CO_2_.

### 2.3. In Vitro Cell Uptake Assay

A cell uptake assay was performed to determine the receptor specificity of [^111^In]In-**12**-**15**.

PC-3 cells were seeded in 12-well plates (2.5 × 10^5^ cells/well) one day prior to the experiment. The next day, cells were washed with Dulbecco’s phosphate-buffered saline (PBS; Gibco) and incubated with 0.5 mL of Kolliphor^®^ HS 15 in PBS (0.06 mg/mL) containing 10^−9^ M [^111^In]In-NeoB (positive control) or [^111^In]In-**12**-**15** in the presence or absence of 10^−6^ M unlabeled NeoB for 1 h at 37 °C. After incubation, the medium was removed, and cells were washed twice with cold (4 °C) PBS. The cells were then exposed to 50 mM glycine (pH 2.8) for 10 min at RT to determine the membrane-bound fraction and lysed using 1M NaOH for >20 min at RT to collect the internalized fractions.

On the day of the experiment, 1 × 10^6^ NCI-H69 cells were incubated with 1.0 mL of the incubation solutions described above. After incubation, the cells were pelleted twice through centrifugation (5 min, 0.5 g, 4 °C) and washed in between with cold (4 °C) PBS.

The collected PC-3 cell fractions (membrane-bound and internalized fractions separately) and NCI-H69 cell pellets (total binding) were measured in a γ-counter (Perkin Elmer, Waltham, MA, USA) to determine the radioactivity uptake. To calculate the amount of radioactivity added to the cells, samples of the incubation solutions were also measured. The results of the uptake assays are expressed as the percentage of added activity (%AA). Data represent the mean ± standard deviation (SD) of triplicate wells.

### 2.4. Animal Model and Experimental Design In Vivo Studies

All animal experiments were approved by the animal welfare committee of the Erasmus MC and were conducted in agreement with the institutional guidelines. In vivo studies were performed to determine (1) the biodistribution profile of [^111^In]In-**12**-**15** and to select the most suitable probe and optimal time point for imaging, (2) to determine the ideal dose of the probe selected based on (1) and (3) to determine the clinical potential of the selected probe in a proof-of-concept simulated image-guided surgery setting.

Five- to eight-week-old male Balb/c nu/nu-specific and opportunistic pathogen-free mice (Janvier Labs, Le Genest-Saint-Isle, France) were used for the biodistribution (*n* = 3/4 mice per condition) and dose optimization studies (*n* = 4 mice per dose), and six-week-old male NMRI-Foxn1 nu/nu mice (Janvier Labs) were used for the proof-of-concept study (*n* = 2). Upon arrival, mice were housed in individually ventilated cages (2–4 mice per cage) with ad libitum access to water and food, and left to acclimate for one week. Animals were subcutaneously inoculated on the right shoulder with PC-3 cells (5 × 10^6^ cells suspended in 200 µL of 1/3 Matrigel (Corning Inc., Corning, NY, USA) and 2/3 Hanks’ balanced salt solution (HBSS; Gibco)). For the biodistribution and proof-of-concept studies, in addition to the PC-3 xenografts, mice were xenografted with NCI-H69 cells on the left shoulder (5 × 10^6^ cells suspended in 200 µL of 1/3 Matrigel and 2/3 HBSS). PC-3 and NCI-H69 xenografts were allowed to grow for 3 and 2.5 weeks, respectively. At the start of the experiment, the tumor sizes were 401 ± 84 mm^3^ and 282 ± 102 mm^3^ for PC-3 and NCI-H69 xenografts, respectively, for the biodistribution study and 378 ± 168 mm^3^ for PC-3 xenografts for the dose optimization study. The tumor sizes were 163 and 409 mm^3^ for PC-3 xenografts and 1066 and 697 mm^3^ for NCI-H69 xenografts for animal 1 and 2, respectively, in the proof-of-concept study.

### 2.5. Administration of [^111^In]In-**12**-**15**

The biodistribution profile of [^111^In]In-**12**-**15** was studied after the administration of approximately 20 MBq/1 nmol. For the dose optimization study, three different doses of [^111^In]In-**14** were administered: 0.75, 1.25 or 1.75 nmol (20 MBq per administered dose). The simulated image-guided surgery was performed after the injection of 20 MBq/0.75 nmol [^111^In]In-**14**. The injected solutions were prepared in PBS containing Kolliphor^®^ HS 15 (0.06 mg/mL). All mice were intravenously injected via the tail vein with a total volume of 200 µL.

### 2.6. In Vivo SPECT/CT/OI for the Biodistribution and Dose Optimization Studies

For the biodistribution study, each dual-modality probe was evaluated at 4 and 24 h post injection (p.i.). In the dose optimization study, each administered dose was studied at 24 h p.i. Whole-body single-photon emission computerized tomography (SPECT)/computed tomography (CT) and optical imaging (OI) were executed for one mouse per group under isoflurane/O_2_ anesthesia using the VECTor^5^/OI-CT small animal scanner (MILabs B.V., Utrecht, The Netherlands). SPECT imaging was performed over 30 min in list mode using the XXUHS-M collimator with a 3.0 mm pinhole diameter. Corresponding CT scans were obtained within 5 min with the following settings: full angle scan, 50 kV tube voltage, 0.21 mA tube current, 500 µm aluminum filter. Lastly, optical imaging was performed with a 624 nm excitation and 692 nm emission filter with 4 × 4 binning for 400 ms or 600 ms in the biodistribution and dose optimization study, respectively.

SPECT images were reconstructed utilizing the similarity-regulated OSEM algorithm (MILabs Rec 12.00 software) with a voxel size of 0.8 mm^3^, 128 subsets, 9 iterations and photo peak energy windows of 171 keV ± 20% and 246 keV ± 20% for indium-111. Two adjacent background windows per photo peak were used for triple-energy window scatter and crosstalk correction. A post-reconstruction Gaussian filter of 1 mm FWHM was applied to both the SPECT and OI images. The CT was reconstructed at 200 µm^3^ and analyzed together with the registered attenuation-corrected SPECT images using PMOD (PMOD 3.9, Zurich, Switzerland). OI images were processed in MILabs OI Post Processing software v2.3.5.

### 2.7. Ex Vivo Biodistribution and Optical Imaging

Ex vivo biodistributions were performed to determine the radioactivity and fluorescent tumor and organ uptake of [^111^In]In-**12**-**15** at 4 and/or 24 h p.i. Blood was collected via a retro-orbital puncture under isoflurane/O_2_ anesthesia, after which mice were euthanized via cervical dislocation. The tumors and organs of interest (adrenal glands, bone, cecum, colon, heart, kidneys, liver, lungs, muscle, pancreas, prostate, small intestine, spleen, stomach and tail) were excised, washed in PBS and dried. The stomach, cecum and intestines were cleared of their contents. The PC-3 and NCI-H69 tumors were cut in half and one half of each tumor was snap-frozen in liquid nitrogen for further ex vivo analysis. The other half of the tumors, together with the bone, kidneys, liver, lungs, muscle, pancreas, small intestine, spleen and stomach were placed in small Petri dishes, and ex vivo optical imaging was performed using the IVIS Spectrum system (Perkin-Elmer) with the following settings: FOV 12.6 cm, medium binning, f-stop 2, 0.5–0.75 s exposure with an excitation/emission filter of 640 nm/680 nm. The fluorescent signal was quantified by drawing a region of interest around the organ/tissue using Living Image version 4.5.2 or 4.7.3 software (Perkin Elmer) and expressed as radiant efficiency [(photons/second/cm^2^/steradian)/(μW/cm^2^)].

After imaging, all tissues and organs were weighed and measured in a γ-counter. To calculate the total radioactivity injected per animal, aliquots of the injected solutions were measured as well. For the adrenal glands and prostate, the average weight of all animals was used because of the low weight of these organs. The percentage injected activity per gram (%IA/g) was determined for each organ and corrected for the %IA present at the injection site (tail).

### 2.8. Ex Vivo Analysis of Xenografts

Fresh frozen xenograft samples were sectioned (10 µm thick) using the CryoStar NX70 cryostat (Thermo Scientific, Waltham, MA, USA) and mounted on glass slides. On consecutive sections, we performed autoradiography to localize the radioactive signal within the samples, a fluorescent scan to localize the fluorescent signal within the samples and hematoxylin-eosin (H&E) staining following standard protocol for histological evaluation. The radioactive slides were covered with copper tape on the back and imaged using the Beaquant system (Ai4r, Nantes, France). The fluorescent slides were scanned using the Odyssey flatbed scanner system (Li-Cor, Lincoln, NE, USA) applying a 700 nm laser. High-resolution images of the H&E-stained sections were acquired using the NanoZoomer digital slide scanner (Hamamatsu Photonics, Shizuoka, Japan).

### 2.9. In Vitro Dead/Alive Cell Binding Assay

A dead/alive cell binding assay was performed to illustrate that dead cell binding was mediated by the sCy5 dye. PC-3 cells were seeded in 24-wells plate (1.25 × 10^5^ cells/well) one day prior to the experiment. The next day, cells were washed with PBS and then either kept alive or killed with 50 µL 70% EtOH solution. Dead and alive cells were incubated with 0.5 mL of 10^−9^ M [^111^In]In-**14** or [^111^In]In-NeoB (control) with or without 10^−6^ M unlabeled NeoB in PBS containing Kolliphor^®^ HS 15 (0.06 mg/mL) for 1 h at 37 °C. Empty wells were included as well to determine the non-specific binding of the probe. After three gentle washes with PBS, the plates were placed on super-resolution phosphor screens for 24 h and read using the Cyclone Plus system (Perkin Elmer).

### 2.10. In Vivo Proof-of-Concept Image-Guided Surgery

SPECT/CT images were acquired at 24 h p.i. while mice (*n* = 2) were under isoflurane/O_2_ anesthesia as described above. After imaging, the mice were sacrificed via cervical dislocation and the PC-3 tumor was removed post-mortem under fluorescent guidance. Fluorescent scans were performed before, during and after tumor excision using the IVIS Spectrum system (Perkin Elmer) with the following settings: FOV 18.6 cm, medium binning, f-stop 2. An image sequence was acquired using a 605 nm excitation filter and emission filters from 660 to 700 nm in 20 nm increments, and a 640 nm excitation filter and emission filters from 680 to 740 nm in 20 nm increments (exposure time: 3–20 s). Images were processed using Living Image software, version 4.7.3. Spectral unmixing was performed to remove tissue autofluorescence.

### 2.11. Ex Vivo Autoradiography on Human Cancer Specimens

An in vitro autoradiography study on human cancer specimens was performed to illustrate the tumor-targeting capacity of **14** for various GRPR-expressing malignancies. This study adhered to the Code of Conduct of the Dutch Federation of Medical Scientific Societies. Fresh frozen human breast cancer, prostate cancer and gastro-intestinal stromal tumor (GIST) specimens (*n* = 5 per cancer type) were obtained from the Erasmus MC tissue bank. Specimens were sectioned at 10 µm thickness and subsequently mounted on glass slides. Tissue sectioning exposed intracellular proteins to which sCy5 could potentially bind. Therefore, tissue sections were incubated with 100 µL of 10^−9^ M [^111^In]In-NeoB in the absence and presence of 10^−5^ M **14** in PBS containing Kolliphor^®^ HS 15 (0.06 mg/mL) for 1 h at RT instead of the other way around. This allowed the focus to only be on the tumor-targeting capacity of **14**. Following incubation, each slide was drained off and washed twice in cold (4 °C) wash buffer containing BSA (167 mM Tris-HCl, 5 mM MgCl_2_, 0.25% BSA), then once in cold wash buffer without BSA and, finally, briefly in cold demi-water. Dried slides were covered with copper tape on the back and imaged with the Beaquant system to localize the radioactive signal within the sample. Adjacent tissue sections were used for H&E staining following standard protocol and imaged using the NanoZoomer digital slide scanner (Hamamatsu Photonics). Tumor regions were manually drawn by experienced pathologists.

### 2.12. Statistics

All statistical analyses were carried out using GraphPad Prism software, version 9 (GraphPad Software Inc., San Diego, CA, USA). A *p* value below 0.05 was considered statistically significant. To confirm receptor specificity, an independent *t*-test was performed for each probe separately to compare total binding to non-specific binding. Regarding the animal studies, significant outliers were detected with Grubbs’ test and excluded from the data set. Tissue and organ uptake of the four dual-modality probes and of the three tested doses were compared by performing a two-way analysis of variance with a Tukey test to correct for multiple comparisons. All data are represented as mean ± standard deviation (SD).

## 3. Results

### 3.1. In Vitro Characterization

In our previous publication, we showed that the binding affinity of the dual-modality probes was in the nanomolar range (IC_50_: 44.1–118.7 nM) [18]. To further characterize **12**-**15** in vitro, an internalization assay was performed. The dual-modality probes presented lower total binding and less receptor-antagonistic properties than the established GRPR-targeting radioligand NeoB; the membrane-bound fraction of [^111^In]In**-12**-**15** was lower than that of [^111^In]In-NeoB (mean of 47.7–61.0% versus 88.0% for [^111^In]In-**12**-**15** and [^111^In]In-NeoB, respectively). In addition, the receptor specificity of **12**-**15** was demonstrated, as an excess of unlabeled NeoB successfully blocked the uptake of [^111^In]In**-12**-**15** in GRPR-positive PC-3 cells (*p* < 0.05) (Figure S1A). An uptake assay using GRPR-negative NCI-H69 cells showed no specific binding for all probes, proving the suitability of using an NCI-H69 xenograft as the negative control for subsequent in vivo studies (Figure S1B).

### 3.2. In Vivo Comparison of the Biodistribution Profiles

**Figure 2:**
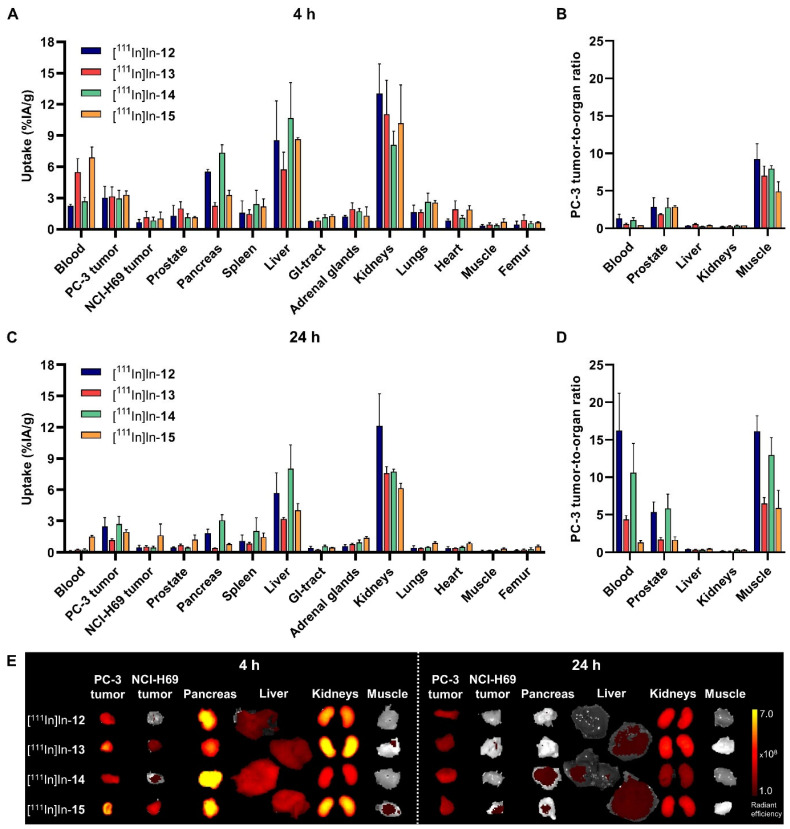
Ex vivo biodistribution of [^111^In]In-**12**-**15** (20 MBq/1 nmol) in mice bearing GRPR-positive PC-3 and GRPR-negative NCI-H69 xenografts. Radioactivity uptake values are expressed as percentage injected activity per gram of tissue (%IA/g) and as PC-3 tumor-to-organ ratios at 4 h (**A**,**B**) and 24 h (**C**,**D**) post injection, and represent the mean ± standard deviation (*n* = 3/4). Ex vivo merged photograph and fluorescence images of a subset of dissected tissues and organs (**E**) are shown for one representative mouse per group. The fluorescent signal is displayed as average radiant efficiency in p/sec/cm^2^/sr per μW/cm^2^.

The first in vivo study examined the impact of linker structures on the biological behavior of the dual-modality probes. Figure 2 depicts the biodistribution of [^111^In]In-**12**-**15** in PC-3 and NCI-H69 tumor-bearing mice at 4 and 24 h p.i.

In spite of their different chemical structures, radioactivity uptake in the PC-3 tumor was similar for all dual-modality probes at the early time point (3.03 ± 1.08%IA/g for [^111^In]In-**12**, 3.16 ± 0.91%IA/g for [^111^In]In-**13**, 2.96 ± 0.78%IA/g for [^111^In]In-**14** and 3.31 ± 0.37%IA/g for [^111^In]In-**15**) (Figure 2A, Table S1). Interestingly, [^111^In]In-**12** and [^111^In]In-**14** showed better tumor retention properties, as tumor uptake was maintained at 24 h p.i. (Figure 2C, Table S2). This contributed to more favorable tumor-to-organ ratios (T/O) for [^111^In]In-**12** and [^111^In]In-**14** compared to [^111^In]In-**13** and [^111^In]In-**15** at the late time point, with a T/O for blood of >10, for the prostate of >5 and for muscle of >12 (*p* < 0.05) (Figure 2B,D). In addition to a faster tumor washout, [^111^In]In-**13** and [^111^In]In-**15** also demonstrated prolonged blood circulation, as the blood uptake at 4 h p.i. was higher than for [^111^In]In-**12** and [^111^In]In-**14** (*p* < 0.01), and was still elevated for [^111^In]In-**15** at 24 h p.i.

High radioactivity uptake levels were also observed in the GRPR-expressing pancreas and the organs responsible for excretion, i.e., the liver and the kidneys. The pancreatic uptake of [^111^In]In-**12** and [^111^In]In-**14** was significantly higher than [^111^In]In-**13** at both time points. Regarding excretion, [^111^In]In-**13** showed significantly less hepatic excretion than the other dual-modality probes at 4 h p.i. (*p* < 0.05). At the late time point, this was the case for both [^111^In]In-**13** and [^111^In]In-**15** (*p* < 0.01). Excretion also largely occurred via the kidneys with [^111^In]In-**12** and [^111^In]In-**13** showing higher kidney uptake than [^111^In]In-**14** at 4 h p.i. (*p* < 0.01). At 24 p.i., kidney uptake was still higher for [^111^In]In-**12** than for all other dual-modality probes (*p* < 0.0001).

In Figure 2E, the distribution of the fluorescent signal in a subset of organs is visualized. This distribution largely corresponds with the distribution of the radioactive signal. Semi-quantitative measurements of the fluorescent intensity verified the highest uptake in the PC-3 tumor, pancreas and kidneys at both time points (Figure S2, Tables S3 and S4). Although, again, a high initial uptake was detected in the pancreas, it also showed a relative rapid washout compared to the PC-3 tumor; a 2.0- vs. 10.9-fold for [^111^In]In-**12**, a 3.7- vs. 5.8-fold for [^111^In]In-**13**, a 2.0- vs. 9.7-fold for [^111^In]In-**14** and a 2.2- vs. 6.0-fold lower uptake for [^111^In]In-**15** in the tumor and pancreas, respectively, measured at 24 vs. 4 h p.i. The longer blood circulation of [^111^In]In-**13** and [^111^In]In-**15** was reflected by a higher signal in background tissues, such as the NCI-H69 tumor and muscle. However, co-localization of the fluorescent and radioactive signal was not observed for the liver, which can be at least partially explained by signal attenuation due to the size of this organ. In addition, the differences between the dual-modality probes were less pronounced for, e.g., the PC-3 tumor at 24 h p.i., which is probably due to the limited sensitivity of this quantification method.

**Figure 3:**
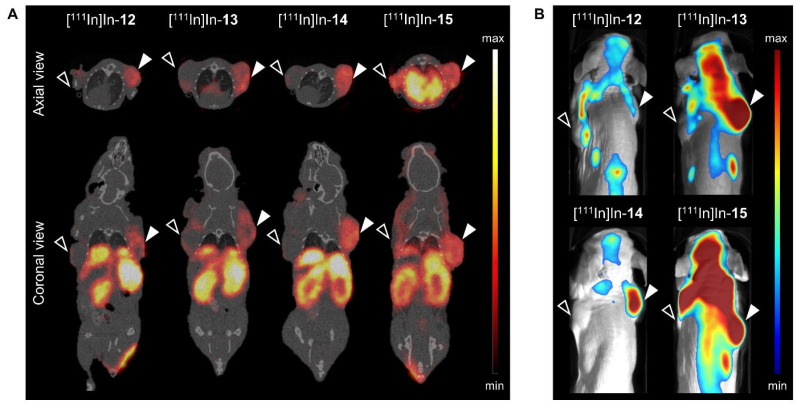
In vivo SPECT/CT (**A**) and optical (**B**) images of GRPR-positive PC-3 (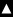; right shoulder) and GRPR-negative NCI-H69 (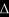; left shoulder) tumor-bearing mice at 24 h post injection of [^111^In]In-**12**-**15** (20 MBq/1 nmol). SPECT/CT images represent an overlay of a CT slice and the corresponding SPECT slice on which the tumor cross-sections are clearly visible. The arrow heads point to the location of the tumor.

Regarding the imaging time point, imaging at 24 h led to improved tumor contrast due to the clearance from normal organs. Figure 3 shows that PC-3 xenografts, unlike NCI-H69 xenografts, could be clearly delineated in both nuclear and optical images at 24 h p.i. (provided that the tumor was located in the field of view). Target specificity was demonstrated by the low signal detected in the GRPR-negative tumor. The signal in the abdominal regions in the coronal SPECT/CT images reflects the excretion of the probes, and the signal in the cardiac region in the axial SPECT/CT image and the increased background signal in the optical image of [^111^In]In-**15** again highlight the prolonged blood circulation of this probe.

Together, these results show that the use of dual-modality probes with a *p*ADA linker in their backbone leads to a higher T/O favorable for good tumor delineation in an image-guided surgery application. Although the biodistribution profiles of [^111^In]In-**12** and [^111^In]In-**14** were largely similar, [^111^In]In-**14** presented lower kidney uptake than [^111^In]In-**12** at both time points. Therefore, [^111^In]In-**14** was selected for further evaluations.

### 3.3. Dose Optimization

**Figure 4:**
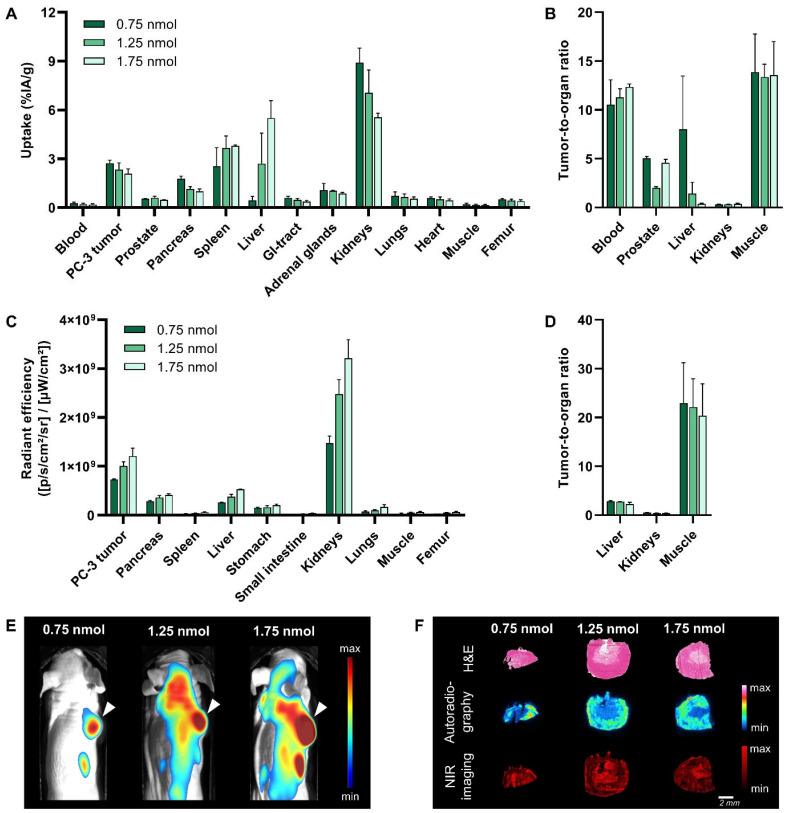
Evaluation of 0.75 nmol, 1.25 nmol and 1.75 nmol [^111^In]In-**14** (20 MBq per administered dose) in PC-3 xenografted mice at 24 h post injection. Ex vivo biodistribution presented as radioactivity uptake (expressed as percentage injected activity per gram of tissue (%IA/g)) (**A**) and the corresponding tumor-to-organ ratios (**B**). Quantification of ex vivo fluorescence imaging of a selection of dissected organs and tissues expressed as average radiant efficiency in p/sec/cm^2^/sr per μW/cm^2^ (**C**) and corresponding tumor-to-organ ratios (**D**). Data are presented as mean ± standard deviation (*n* = 4). In vivo whole-body optical imaging displayed as merged photographs and fluorescence images (**E**). The arrow head points at the location of the PC-3 tumor. Ex vivo PC-3 xenograft analysis of one representative mouse per group demonstrating the histology (top row), radioactive signal distribution (middle row) and fluorescent signal localization (bottom row) (**F**).

The purpose of the next in vivo study was to study the effect of the injected dose on T/O to select the ideal dose to achieve maximum image contrast with both imaging modalities. Figure 4 presents the evaluation of three different doses, i.e., 0.75 nmol, 1.25 nmol and 1.75 nmol, of [^111^In]In-**14** in PC-3 xenografted mice at 24 h p.i.

With respect to nuclear imaging, no clear differences were observed in the SPECT/CT images (Figure S3), but a slightly lower radioactivity uptake in the tumor with increasing dose was recognized (2.74 ± 0.18%IA/g, 2.35 ± 0.40%IA/g and 2.09 ± 0.30%IA/g for 0.75, 1.25 and 1.75 nmol, respectively) (Figure 4A, Table S5). However, significant changes were noted in the route of excretion. The injection of a higher dose resulted in reduced kidney uptake (8.90 ± 0.91%IA/g, 7.05 ± 1.40%IA/g and 5.55 ± 0.24%IA/g for 0.75, 1.25 and 1.75 nmol, respectively) (*p* < 0.001). In contrast, a significantly higher radioactivity uptake in the liver and spleen was observed with an increasing dose. This indicates that hepatic clearance plays a greater role than renal clearance in the excretion of [^111^In]In-**14** when higher doses are administered. The increased hepatic excretion translated into a 20.5-fold lower tumor-to-liver ratio for 1.75 vs. 0.75 nmol (*p* < 0.0001) (Figure 4B).

Regarding optical imaging, the fluorescence images showed a strong increase in the fluorescent signal in the tumor upon application of an increasing dose (Figure 4E). The quantification of this signal revealed a 1.7-fold increase for 1.75 vs. 0.75 nmol (*p* < 0.0001) (Figure 4C, Table S6). However, the fluorescent signal in most background organs also increased, e.g., 2.0-, 2.2- and 1.9-fold higher signal in the liver, kidneys and muscle, respectively, when a dose of 1.75 vs. 0.75 nmol was administered. Together, this resulted in similar T/O for all doses (Figure 4D). Based on this and the better tumor-to-liver ratios of the radioactive signal distribution following the injection of a lower dose, 0.75 nmol was identified as the ideal dose.

Since the radioactive and fluorescent signal distribution in the tumor showed an opposite trend with respect to dose, an ex vivo analysis of PC-3 xenografts was performed to localize the respective signals within the tumor. From the data in Figure 4F, it is apparent that the radioactive and fluorescent signal are heterogeneously distributed throughout the tumor. H&E staining revealed that the radioactive signal is mostly restricted to viable regions, while the fluorescent signal is not restricted and strongest in non-viable regions, i.e., the necrotic core of the tumor. The binding of [^111^In]In-**14** to dead cells was confirmed by an in vitro cell binding experiment (Figure S4). The fact that the binding of [^111^In]In-**14** to dead PC-3 cells, unlike living cells, could not be blocked by an excess of unlabeled NeoB illustrates that the dead cell binding is not mediated by the GRPR binding domain.

### 3.4. Translational Applicability

**Figure 5:**
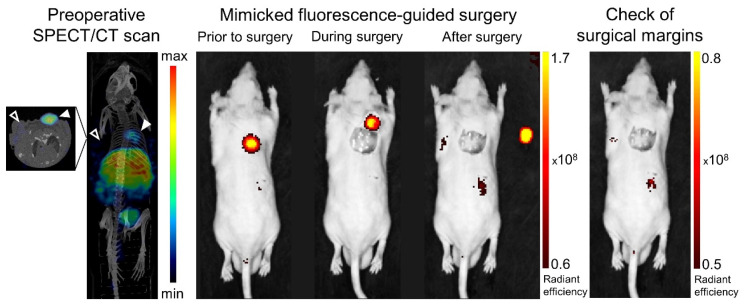
Proof-of-concept image-guided surgery on PC-3 (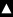; right shoulder) and NCI-H69 (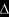; left shoulder) tumor-bearing mice at 24 h post injection 20 MBq/0.75 nmol [^111^In]In-**14**. Shown are a preoperative SPECT/CT scan (**left panel**), post-mortem merged photograph and fluorescence images obtained prior to, during and after PC-3 tumor resection (**middle panel**), plus a final image to check the surgical margins with a more sensitive scale (**right panel**). The fluorescent signal is displayed as average radiant efficiency in p/sec/cm^2^/sr per μW/cm^2^. For the SPECT/CT scan, a maximum intensity projection is shown in combination with an axial SPECT/CT image representing an overlay of a CT slice and the corresponding SPECT slice on which the tumor cross-sections are clearly visible. The arrow heads point to the location of the tumor.

In order to assess the applicability of [^111^In]In-**14** for image-guided tumor resection, image-guided surgery was mimicked on PC-3 and H69 xenografted mice (Figure 5). The GRPR-positive tumor could be clearly localized via a preoperative SPECT/CT evaluation at 24 h p.i. Post-mortem IVIS imaging allowed the location of the incision to be accurately determined and the tumor tissue to be distinguished and monitored during the operative procedure through fluorescent guidance.

**Figure 6:**
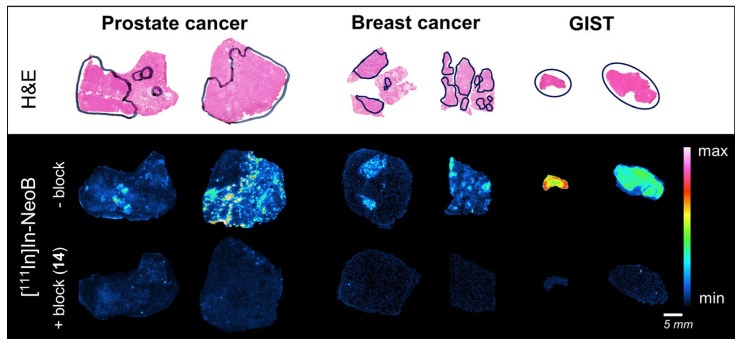
Hematoxylin-eosin staining (**top row**) and ex vivo autoradiography of human prostate cancer, breast cancer and gastro-intestinal stromal tumor (GIST) sections from six representative patients demonstrating binding of [^111^In]In-NeoB in the absence (− block; **middle row**) or presence (+ block (**14**); **bottom row**) of an excess of **14**. Tumor regions were circled in black by experienced pathologists.

An ex vivo binding study was performed on a wide range of solid cancer specimens (i.e., breast cancer, prostate cancer and GIST) to demonstrate the broad applicability of [^111^In]In-**14**. Figure 6 shows that an excess of the dual-modality probe can successfully block the [^111^In]In-NeoB signal in all three cancer types studied, illustrating its potential to bind to various human GRPR-positive cancerous tissues.

## 4. Discussion

Surgery is the gold standard treatment for several solid tumors. Complete tumor resection is related to favorable outcomes, but it can be challenging for the surgeon to delineate tumors intraoperatively solely by visual and tactile guidance. To increase surgical precision, guidance by fluorescence imaging has been proposed to aid in the differentiation of tumor tissue from adjacent non-cancerous tissue [20]. The use of dual-labeled analogs containing both a radioisotope and fluorescent dye can combine the strengths of two modalities; tumor localization by nuclear imaging and real-time visualization by fluorescence imaging. The field of image-guided surgery can build on the success of targeted radioligands, where a common strategy is to add a fluorescent dye to an established radioligand. In a previous publication, we described the development of four dual-modality probes based on the potent radioligand NeoB that targets the GRPR and using two different linkers, i.e., the *p*ADA and PEG_4_ linker [18]. In this study, we focused on characterizing the biological behavior of the four developed GRPR-targeting dual-modality probes, optimizing the dose of the most promising probe and determining its translational applicability.

As a result of the relatively small size of radioligands, the conjugation of a fluorescent dye and linker can greatly influence their biological behavior [21]. We observed that the receptor-specific properties of NeoB were preserved within the developed probes, most likely because the binding domain was unchanged. However, the strong antagonistic properties of NeoB were not retained as the intracellular fraction of the probes was increased by ~35%. We assume that this effect is probably due to the incorporation of the dye rather than the differences in linker structures. The observed increase in cell internalization after dye conjugation has also been reported for the radioligands RM2 and PSMA-11 [22,23]. Moreover, since the use of GRPR antagonists has been shown to lead to higher levels of tumor accumulation, the more agonistic properties of the developed dual-modality probes could partly explain the observed decrease in total binding [24].

The incorporation of the fluorescent dye also largely determines the in vivo biodistribution. From comparing the biodistribution profile of the probes with that of the radiolabeled parent peptide NeoB at similar doses [25], it is apparent that the probes have prolonged blood circulation. The prolonged circulation time, and thus delayed clearance, results in higher kidney uptake at both time points. Moreover, the injection of the probes led to higher levels of liver accumulation. This biodistribution profile is in line with our preliminary data on [^111^In]In-**12** and [^111^In]In-**15** and with the findings of Zhang et al., who characterized a GRPR-targeting dual-modality probe that was based on the radioligand RM2 [18,22]. The observed increase in liver uptake can at least partly be attributed to metabolism of the dye in this organ [26]. Accordingly, a loss of fluorescent signal may be the consequence of the degradation of the dye [27]. This might also be the reason for the discrepancy when it comes to the co-localization of the fluorescent and radioactive signal in the liver. The observed impact of the dye on the biological behavior of probes has previously been reported for other peptide-based dual-modality probes [28,29,30]. As different fluorescent dyes have different chemical properties, further research into the use of other dyes could potentially lead to optimization of the biodistribution profile. Taken together, these findings underline the need for preclinical evaluations when radiolabeled peptides are repurposed for image-guided surgery applications.

The in vivo data also demonstrated that the linker contributed to the biodistribution of the probes. This is in agreement with the findings of Shrivastava et al. [31], who illustrated that fluorescent GRPR-targeting peptides are highly sensitive to linker adaptations. The two linkers evaluated in our study were the *p*ADA linker, which is part of the original NeoB structure, and PEG_4_. PEG_4_ provides more spacing than *p*ADA and was selected to potentially limit steric hindrance from the fluorescent label. The dual-modality probes [^111^In]In-**12** and [^111^In]In-**14**, both with a *p*ADA linker in their backbone, presented with better tumor retention properties. This suggests that the more rigid *p*ADA linker is an integral part of NeoB and partly responsible for the good tumor-targeting properties of this radioligand. [^111^In]In-**13** and [^111^In]In-**15** showed a longer blood circulation time and were mainly cleared via the kidneys, which could be explained by the hydrophilic nature of the PEG_4_ linker located in their backbones [32]. Although [^111^In]In-**12** and [^111^In]In-**13** contain an extra PEG_4_ linker between the lysine and TCO moiety to create more distance between the dye and binding domain, this seemed to have an effect on the clearance pathway rather than binding [22]. Our findings emphasize that the linker selection should be carefully evaluated as it can considerably impact the biological behavior of dual-modality probes.

High image contrast is required for clear tumor delineation in both a preoperative and intraoperative setting. We have shown that adjusting the imaging time point can positively impact image contrast, as delayed imaging benefits from increased clearance. Due to the natural affinity of cyanine dyes for human serum albumin [33], others have also reported on the need for delayed imaging after dye conjugation into the normally rapidly cleared peptides [34]. Twenty-four hours after injection, the dual-modality probe with the most favorable T/O was [^111^In]In-**14**. Although the T/O for most background organs was more than sufficient to distinguish tumor tissue from normal tissue (i.e., >2) [9], uptake in the organs responsible for excretion was still high and would hinder the localization of tumor lesions in their immediate vicinity or downstream of the excretion route. However, it is encouraging that tumor uptake was maintained over time, because it suggests that imaging at an even later time point with further increased clearance might improve contrast in these regions.

Another important factor in achieving maximum image contrast with both imaging modalities is dose. A common challenge is to adapt the dose to match the sensitivity of both imaging techniques [35,36]. Surprisingly, the effect of administering increasing doses was observed best in the clearance pathway; liver uptake was more pronounced. Despite the fact that a surfactant was used during radiolabeling and injection preparation to prevent peptide aggregation and their subsequent accumulation in the liver, we suspect that at higher doses, the solubility limit of our probe was reached [37]. The concomitant decrease in renal clearance is most likely a result of this increased liver uptake. A higher liver uptake is less favorable when aiming for an image-guided surgery application in the abdominal cavity, hence the ideal dose of [^111^In]In-**14** for our preclinical model was 0.75 nmol, as this led to a tumor-to-liver ratio of >2. The translation of our dual-modality probe requires additional studies to investigate whether, for example, higher concentrations of the surfactant can improve solubility.

All three tested doses were coupled to an equal amount of radioactivity, meaning that for higher doses the portion of unlabeled probe (i.e., containing only a fluorescent label) was increased. We observed that administering increasing doses resulted in a slight decrease in the radioactive signal measured in the tumor, indicative of receptor saturation. Incongruous with receptor saturation, tumor fluorescence was actually enhanced. Further analysis revealed strong and GRPR-nonspecific binding of [^111^In]In-**14** to necrotic tumor cores. A possible explanation for this might be that dead cell binding is facilitated by sCy5, as this characteristic of cyanine-dyes has previously been reported (e.g., Xie et al., 2015 [38]; Stroet et al., 2021 [39]). The fact that an increase in the applied dose led to a further increase in the fluorescent tumor signal can be attributed to higher amounts of probe available to bind to dead cells, and to the abundance of cytoplasmic proteins. Since necrosis is a pathologic process and commonly observed in most solid tumors (e.g., breast and GIST tumors), this additional binding would only positively contribute to the differentiation of tumor tissue [40].

In the last part of the study, we provided implications for the clinical applicability of our dual-modality probe [^111^In]In-**14**. The mimicked image-guided surgery demonstrated the ability to clearly visualize the tumor during all surgical steps and again underlined the target specificity of our probe, as NCI-H69 xenografts were not detected. Li et al. [41] also reported on the successful removal of tumor tissue by using a GRPR-targeting dual-modality probe for fluorescent guidance in their orthotopic brain tumor mouse model. Furthermore, we were able to establish the applicability of **14** for the detection and delineation of human prostate, breast and GIST tumors. Together, these findings indicate that our developed dual-modality probe would be a promising candidate for clinical translation. To develop a full picture of the translational potential of our dual-modality probe, additional studies investigating tumor retention over a longer period of time and up to the moment of surgery are needed.

## 5. Conclusions

In this study, we described the preclinical characterization of four dual-modality probes for preoperative nuclear imaging and intraoperative fluorescence imaging of GRPR-positive solid tumors. We demonstrated that the probes with a *p*ADA linker in their backbone presented the best tumor retention properties and that the addition of a PEG_4_ linker negatively influenced the clearance rate. The image contrast was improved by delaying the imaging time point due to increased clearance, but the ideal time point with respect to renal clearance may not yet have been reached. The tumor-to-organ ratios of the most promising probe were also increased by reducing the injected dose to circumvent receptor saturation and reduce liver uptake. The binding of sCy5 to necrotic areas further enhanced the fluorescent signal. Finally, the ex vivo binding to various human cancer tissues and the ability to clearly visualize the tumor in a simulated surgical setting underline the potential for future clinical translation of our developed probe for image-guided surgery of GRPR-positive tumors.

## Data Availability

The data presented in this study are available on request from the corresponding author.

## Supplementary Materials

The following supporting information can be downloaded at: https://www.mdpi.com/article/10.3390/cancers15072161/s1. Methods: Chemistry [42]; Figure S1: Uptake of [^111^In]In-**12**-**15** and [^111^In]In-NeoB (positive control) by GRPR-positive PC-3 cells (A) and GRPR-negative NCI-H69 cells (B) at 1 h after incubation with 10^−9^ M (20 MBq/nmol); Figure S2: Quantification of ex vivo fluorescence imaging of a selection of dissected organs and tissues at 4 h (A) and 24 h (B) post injection of [^111^In]In-**12**-**15** (20 MBq/1 nmol); Figure S3: SPECT/CT images of PC-3 xenografted mice at 24 h post injection of 0.75, 1.25 or 1.75 nmol of [^111^In]In-**14** (20 MBq per administered dose); Figure S4: Visualization of the radioactive signal in a well plate containing dead and alive PC-3 cells incubated with 10^−9^ M [^111^In]In-**14** or [^111^In]In-NeoB (control) (20 MBq/nmol); Table S1: Biodistribution of [^111^In]In-**12**-**15** (20 MBq/1 nmol) in PC-3 and NCI-H69 xenografted balb/c nu/nu mice at 4 h post injection; Table S2: Biodistribution of [^111^In]In-**12**-**15** (20 MBq/1 nmol) in PC-3 and NCI-H69 xenografted balb/c nu/nu mice at 24 h post injection; Table S3: Organ/tissue fluorescence after dissection of PC-3 and NCI-H69 xenografted balb/c nu/nu mice at 4 h post injection of [^111^In]In-**12**-**15** (20 MBq/1 nmol); Table S4: Organ/tissue fluorescence after dissection of PC-3 and NCI-H69 xenografted balb/c nu/nu mice at 24 h post injection of [^111^In]In-**12**-**15** (20 MBq/1 nmol); Table S5: Biodistribution of 0.75, 1.25 or 1.75 nmol [^111^In]In-**14** (20 MBq per administered dose) in PC-3 xenografted balb/c nu/nu mice at 24 h post injection; Table S6: Organ/tissue fluorescence after dissection of PC-3 xenografted balb/c nu/nu mice at 24 h post injection of 0.75, 1.25 or 1.75 nmol [^111^In]In-**14** (20 MBq per administered dose).
