# Supplementary material for: Pre- and Intraoperative Visualization of GRPR-Expressing Solid Tumors: Preclinical Profiling of Novel Dual-Modality Probes for Nuclear and Fluorescence Imaging
Source: Cancers (Basel). 2023 Apr 5;15(7):2161. doi: 10.3390/cancers15072161 (PMC10093582; doi:10.3390/cancers15072161)
Supplement: Supplementary file 1 [file cancers-15-02161-s001.zip › cancers-2213544-supplementary.pdf]

Supplementary Materials

Methods

Chemistry

The four dual-modality NeoB analogs were synthesized following the Fmoc solid phase peptide synthesis strategy. The amino acids (4.0 equiv.) were coupled to the solid support using standard coupling agents, 2-(1*H*-benzotriazol-1-yl)-1,1,3,3-tetramethyluronium hexafluorophos-phate and Oxyma Pure (3.9 and 4.0 equiv., respectively), under basic conditions (pH ~9) for 1 h at RT. Using the same conditions, the linkers (Fmoc-NH-PEG_4-COOH or Fmoc-NH-*p*ADA-OH) (2.0 equiv.) and Fmoc-L-Lys(Boc)-OH (4.0 equiv.) were coupled for 2 h at RT and then the Fmoc protecting group was removed with a solution of 20% piperidine in dimethylformamide for 15 min at RT. The DOTA chelator (3.0 equiv.) was introduced using benzotriazole-1-yl-oxy-tris-pyrrolidino-phosphonium hexafluorophosphate (PyBOP; 3.0 equiv.) under basic conditions (pH ~9); coupling was performed for 2 h at RT. A cocktail of 1,1,1,3,3,3-hexafluoro-2-propanol/dichloromethane (20:80 V/V) was used to cleave the peptides from the solid support for 1 h at RT. Coupling of the 4-amino-2,6-dimethylheptane (2.5 equiv.) on the C-terminus was performed using PyBOP (2.5 equiv.) under basic conditions (pH ~9). A trifluoroacetic acid (TFA)/water/triisopropyl silane cocktail (95:2.5:2.5 V/V/V) was then used to remove the side chain protecting groups for 1 h at RT. Due to the sensitivity of the TCO to acidic conditions, excess TFA was removed by C-18 Sep-Pak purification prior to coupling of TCO-NHS ester or TCO-PEG_4-NHS ester (3.0 equiv.) in water/acetonitrile (1:1 V/V). Finally, the tetrazinyl-fluorescent dye, Tz-sCy5 (1.1 equiv.), was conjugated to the TCO-functionalized peptides via the inverse electron-demand Diels-Alder reaction to obtain the final dual-modality probes **12**, **13**, **14** and **15** [1].

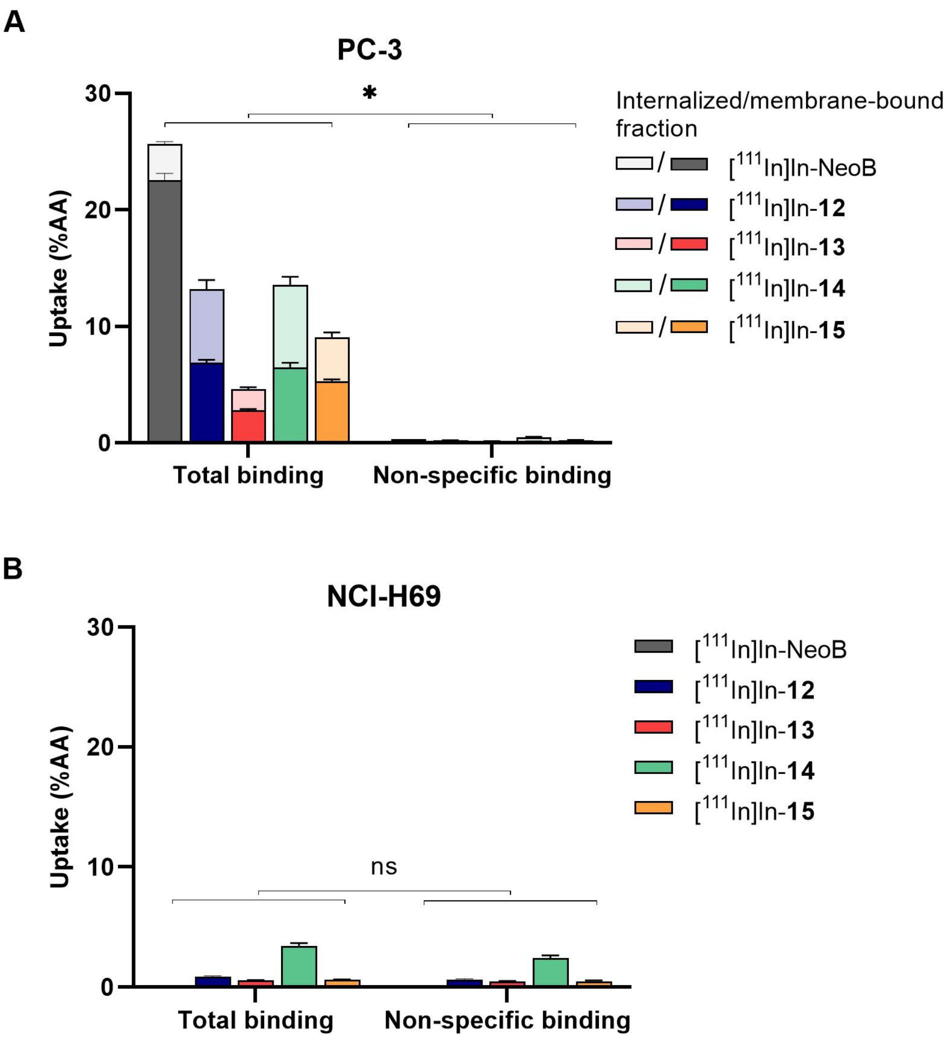

Figure S1. Uptake of $[^{111}In]In$-**12-15** and $[^{111}In]In$-NeoB (positive control) by GRPR-positive PC-3 cells (A) and GRPR-negative NCI-H69 cells (B) at 1 h after incubation with 10^{-9} M (20 MBq/nmol). The uptake by PC-3 cells is split into the internalized and membrane-bound fraction. Non-specific binding is defined as the uptake in the presence of a 1000x excess of unlabeled NeoB. Uptake values are expressed as percentage added activity (%AA). Results shown represent data from one independent experiment performed in triplicate (mean ± standard deviation). * = $p < 0.05$, ns = not significant.

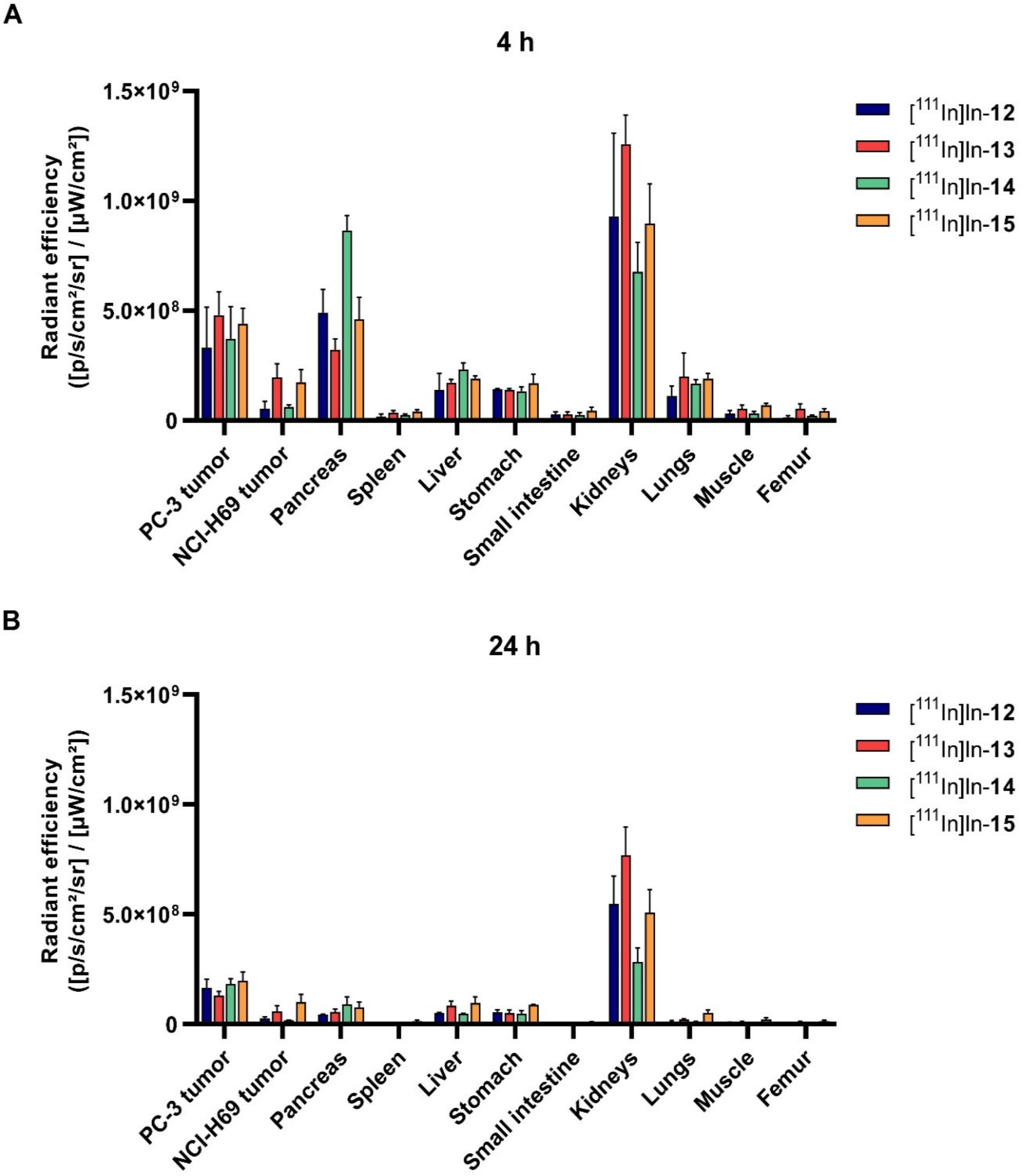

Figure S2. Quantification of *ex vivo* fluorescence imaging of a selection of dissected organs and tissues at 4 h (A) and 24 h (B) post injection of $[^{111}In]In$-**12-15** **(**20 MBq/1 nmol). Data are presented as mean ± standard deviation (n = 3/4).

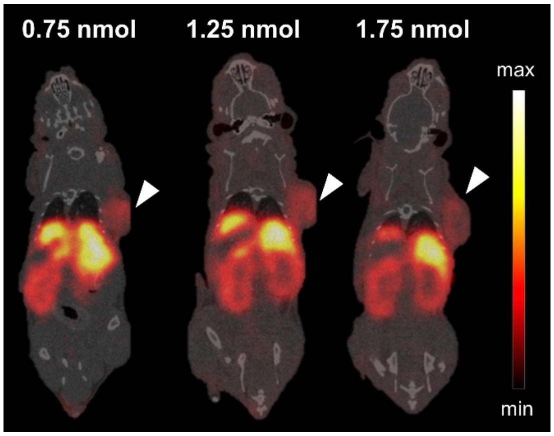

Figure S3. SPECT/CT images of PC-3 xenografted mice at 24 h post injection of 0.75, 1.25 or 1.75 nmol of [^{111}In]In-**14** (20 MBq per administered dose). SPECT/CT images represent an overlay of a CT slice and the corresponding SPECT slice on which the tumor cross-section is clearly visible. The arrow head points at the location of the tumor.

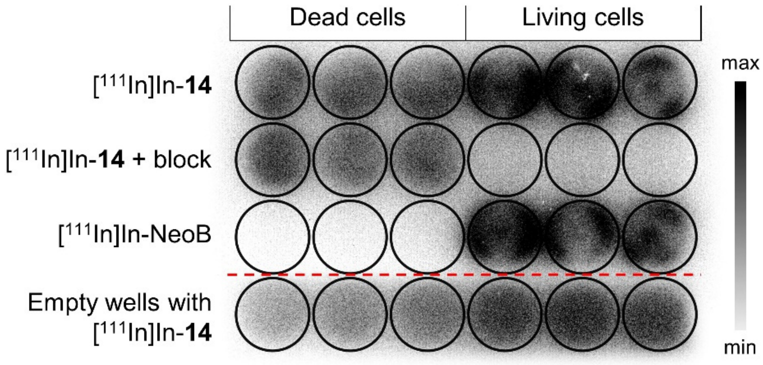

Figure S4. Visualization of the radioactive signal in a well-plate containing dead and alive PC-3 cells incubated with 10^{-9} M [^{111}In]In-**14** or [^{111}In]In-NeoB (control) (20 MBq/nmol). Blocking was performed by co-incubation with 10^{-6} M unlabeled NeoB. The wells below the red dotted line were empty and show the non-specific binding to plastic.

Table S1. Biodistribution of [^{111}In]In-**12-15** (20 MBq/1 nmol) in PC-3 and NCI-H69 xenografted balb/c nu/nu mice at 4 h post injection. Uptake values are expressed as percentage injected activity per gram of tissue (%IA/g) and represent the mean ± standard deviation. The asterisk shows the significance with * $p < 0.05$. $^{12/13/14/15}$ significant difference as compared with probe [^{111}In]In-**12-15** for the same organ. ^ Outlier excluded from dataset based on Grubbs' test ($n = n - 1$).

**Organ/tissue**	[^{111}In]In-**12** (n = 3)	[^{111}In]In-**13** (n = 4)	[^{111}In]In-**14** (n = 4)	[^{111}In]In-**15** (n = 3)
Blood	2.26 ± 0.12^{*13,15}	5.49 ± 1.27^{*12,14}	2.68 ± 0.38^{*13,15}	6.91 ± 0.98^{*12,14}
PC-3 tumor	3.03 ± 1.08	3.16 ± 0.91	2.96 ± 0.78	3.31 ± 0.37
NCI-H69 tumor	0.68 ± 0.25	1.16 ± 0.56^	0.84 ± 0.32	1.03 ± 0.61
Prostate	1.30 ± 0.98	2.00 ± 0.65	1.14 ± 0.34	1.15 ± 0.10
Pancreas	5.53 ± 0.21^{*13}	2.25 ± 0.31^{*12,14}	7.35 ± 0.75^{*13,15}	3.28 ± 0.47^{*14}
Spleen	1.59 ± 1.13	1.45 ± 0.42	2.34 ± 1.31	2.18 ± 0.74
Liver	8.55 ± 3.77^{*13}	5.75 ± 1.64^{*12,14,15}	10.69 ± 3.40^{*13}	8.66 ± 0.13$^{\wedge,*13}$
Stomach	1.00 ± 0.26	1.03 ± 0.31	1.25 ± 0.29	1.47 ± 0.28
Small intestine	1.17 ± 0.32	0.76 ± 0.35	1.21 ± 0.40	1.79 ± 1.15
Caecum	0.81 ± 0.27	0.81 ± 0.23	1.03 ± 0.15	1.08 ± 0.05
Colon	0.76 ± 0.004^	0.67 ± 0.11	1.31 ± 0.22	1.25 ± 0.14
Adrenal glands	1.22 ± 0.10	1.95 ± 0.60	1.73 ± 0.27	1.30 ± 0.85
Kidneys	13.04 ± 2.83^{*14,15}	11.05 ± 3.27^{*14}	8.11 ± 1.30^{*12,13}	10.19 ± 3.68^{*12}
Lungs	1.64 ± 0.68	1.65 ± 0.19^	2.63 ± 0.85	2.55 ± 0.22
Heart	0.84 ± 0.14	1.93 ± 0.79	1.11 ± 0.20	1.89 ± 0.39
Muscle	0.33 ± 0.11	0.46 ± 0.15	0.38 ± 0.11	0.72 ± 0.29
Femur	0.46 ± 0.31	0.91 ± 0.48	0.58 ± 0.16	0.65 ± 0.07
Tumor-to-organ ratio				
PC-3 tumor-to-blood	1.35 ± 0.53	0.58 ± 0.12	1.11 ± 0.29	0.42 ± 0.001^
PC-3 tumor-to-prostate	2.84 ± 1.24	1.88 ± 0.07^	2.79 ± 1.23	2.88 ± 0.16
PC-3 tumor-to-liver	0.36 ± 0.03	0.56 ± 0.09	0.28 ± 0.03	0.40 ± 0.02^
PC-3 tumor-to-kidneys	0.23 ± 0.05	0.30 ± 0.07	0.36 ± 0.04	0.39 ± 0.003^
PC-3 tumor-to-muscle	9.23 ± 2.05^{*13,15}	7.00 ± 1.31^{*12,15}	7.96 ± 0.40^{*15}	4.92 ± 1.28^{*12,13,14}

Table S2. Biodistribution of [^{111}In]In-**12-15** (20 MBq/1 nmol) in PC-3 and NCI-H69 xenografted balb/c nu/nu mice at 24 h post injection. Uptake values are expressed as percentage injected activity per gram of tissue (%IA/g) and represent the mean ± standard deviation. The asterisk shows the significance with * $p < 0.05$. $^{12/13/14/15}$ significant difference as compared with probe [^{111}In]In-**12-15** for the same organ. ^ Outlier excluded from dataset based on Grubbs' test ($n = n$ - 1).

**Organ/tissue**	[^{111}In]In-**12** (n = 4)	[^{111}In]In-**13** (n = 4)	[^{111}In]In-**14** (n = 4)	[^{111}In]In-**15** (n = 4)
Blood	0.16 ± 0.03^{*15}	0.27 ± 0.04	0.28 ± 0.08	1.52 ± 0.13^{*12}
PC-3 tumor	2.49 ± 0.83^{*13}	1.17 ± 0.13^{*12,14}	2.71 ± 0.72^{*13}	1.97 ± 0.22
NCI-H69 tumor	0.47 ± 0.21	0.55 ± 0.10^	0.47 ± 0.13	1.64 ± 1.08
Prostate	0.46 ± 0.07	0.69 ± 0.12	0.35 ± 0.23	1.24 ± 0.43
Pancreas	1.85 ± 0.38^{*13}	0.42 ± 0.02^{*12,14}	3.07 ± 0.53^{*13,15}	0.79 ± 0.08^{*14}
Spleen	1.10 ± 0.57	0.84 ± 0.13	2.06 ± 1.25	1.48 ± 0.39
Liver	5.70 ± 1.93^{*13,14,15}	3.19 ± 0.16^{*12,14}	8.02 ± 2.29^{*12,13,15}	4.02 ± 0.65^{*12,14}
Stomach	0.46 ± 0.17	0.24 ± 0.02	0.65 ± 0.08	0.44 ± 0.03
Small intestine	0.30 ± 0.13	0.19 ± 0.03	0.50 ± 0.08	0.37 ± 0.04
Caecum	0.41 ± 0.15	0.28 ± 0.03	0.58 ± 0.13	0.46 ± 0.04
Colon	0.50 ± 0.20	0.38 ± 0.06	0.61 ± 0.10	0.63 ± 0.08
Adrenal glands	0.61 ± 0.16	0.79 ± 0.10	0.90 ± 0.22	1.40 ± 0.14
Kidneys	12.14 ± 3.09 ^{*13,14,15}	7.60 ± 0.62^{*12,15}	7.74 ± 0.23^{*12,15}	6.13 ± 0.47^{*12,13,14}
Lungs	0.44 ± 0.19	0.39 ± 0.04^	0.50 ± 0.06	0.91 ± 0.14
Heart	0.40 ± 0.15	0.42 ± 0.03	0.52 ± 0.09	0.87 ± 0.09
Muscle	0.16 ± 0.06	0.18 ± 0.02	0.21 ± 0.04	0.36 ± 0.08
Femur	0.22 ± 0.04^	0.27 ± 0.06	0.33 ± 0.14	0.57 ± 0.14
Tumor-to-organ ratio				
PC-3 tumor-to-blood	16.21 ± 5.02 ^{*13,14,15}	4.37 ± 0.51^{*12,14}	10.59 ± 3.91 ^{*12,13,15}	1.31 ± 0.25^{*12,14}
PC-3 tumor-to-prostate	5.34 ± 1.35^{*13,15}	1.72 ± 0.26^{*12,14}	5.84 ± 1.91$^{\wedge,*13,15}$	1.69 ± 0.39^{*12,14}
PC-3 tumor-to-liver	0.44 ± 0.04	0.37 ± 0.04	0.35 ± 0.06	0.50 ± 0.06
PC-3 tumor-to-kidneys	0.20 ± 0.04	0.16 ± 0.03	0.35 ± 0.10	0.32 ± 0.03
PC-3 tumor-to-muscle	16.14 ± 2.05^{*13,15}	6.48 ± 0.81^{*12,14}	12.95 ± 2.36^{*13,15}	5.88 ± 2.37^{*12,14}

Table S3. Organ/tissue fluorescence after dissection of PC-3 and NCI-H69 xenografted balb/c nu/nu mice at 4 h post injection of [^{111}In]In-**12-15** (20 MBq/1 nmol). The signal is expressed as average radiant efficiency in 10^8 p/sec/cm^2/sr per µW/cm^2. The asterisk shows the significance with * $p < 0.05$. $^{12/13/14/15}$ significant difference as compared with probe [^{111}In]In-**12-15** for the same organ. ^ Outlier excluded from dataset based on Grubbs' test ($n = n - 1$).

**Organ/tissue**	[^{111}In]In-**12** (n = 3)	[^{111}In]In-**13** (n = 4)	[^{111}In]In-**14** (n = 4)	[^{111}In]In-**15** (n = 3)
PC-3 tumor	3.30 ± 1.86	4.80 ± 1.06	3.71 ± 1.47	4.40 ± 0.69
Kidneys	9.31 ± 3.81^{*13,14}	12.59 ± 1.34 ^{*12,14,15}	6.77 ± 1.34^{*12,13,15}	8.97 ± 1.82^{*13,15}
Pancreas	4.91 ± 1.06^{*14}	3.21 ± 0.49^{*14}	8.64 ± 0.69^{*12,13,15}	4.60 ± 1.00^{*14}
Liver	1.38 ± 0.76	1.72 ± 0.15	2.32 ± 0.31	1.91 ± 0.11
NCI-H69 tumor	0.54 ± 0.33	1.95 ± 0.63	0.62 ± 0.08	1.73 ± 0.58
Femur	0.15 ± 0.07	0.53 ± 0.22	0.22 ± 0.03	0.43 ± 0.10
Muscle	0.30 ± 0.15	0.53 ± 0.16	0.32 ± 0.08	0.69 ± 0.08
Spleen	0.19 ± 0.11	0.35 ± 0.01	0.25 ± 0.04	0.41 ± 0.08
Small intestine	0.72 ± 0.12	0.27 ± 0.12	0.26 ± 0.10	0.44 ± 0.16
Lungs	1.12 ± 0.43	1.99 ± 1.07	1.69 ± 0.16	1.90 ± 0.24
Stomach	1.44 ± 0.006^	1.39 ± 0.006^	1.33 ± 0.19	1.69 ± 0.41
Tumor-to-organ ratio				
PC-3 tumor-to-liver	2.39 ± 0.19	2.78 ± 0.46	1.59 ± 0.55	2.30 ± 0.26
PC-3 tumor-to-kidneys	0.35 ± 0.07	0.38 ± 0.04	0.53 ± 0.12	0.49 ± 0.03
PC-3 tumor-to-muscle	10.87 ± 1.45	9.37 ± 1.66	11.48 ± 2.08	6.35 ± 0.31

Table S4. Organ/tissue fluorescence after dissection of PC-3 and NCI-H69 xenografted balb/c nu/nu mice at 24 h post injection of [^{111}In]In-**12-15** (20 MBq/1 nmol). The signal is expressed as average radiant efficiency in 10^8 p/sec/cm^2/sr per µW/cm^2. The asterisk shows the significance with * $p < 0.05$. $^{12/13/14/15}$ significant difference as compared with probe [^{111}In]In-**12-15** for the same organ. ^ Outlier excluded from dataset based on Grubbs' test ($n = n - 1$).

**Organ/tissue**	[^{111}In]In-**12** (n = 4)	[^{111}In]In-**13** (n = 4)	[^{111}In]In-**14** (n = 4)	[^{111}In]In-**15** (n = 4)
PC-3 tumor	1.65 ± 0.39	1.31 ± 0.18	1.83 ± 0.23	1.98 ± 0.41
Kidneys	5.47 ± 1.26^{*13,14}	7.69 ± 1.30^{*12,14,15}	2.83 ± 0.64^{*12,13,15}	5.06 ± 1.07^{*13,14}
Pancreas	0.45 ± 0.01^	0.55 ± 0.14	0.89 ± 0.34	0.77 ± 0.24
Liver	0.49 ± 0.04^	0.84 ± 0.22	0.47 ± 0.03^	0.96 ± 0.28
NCI-H69 tumor	0.26 ± 0.06^{*15}	0.58 ± 0.26	0.16 ± 0.02^{*15}	1.00 ± 0.36^{*12,14}
Femur	0.06 ± 0.007	0.11 ± 0.02	0.06 ± 0.003	0.13 ± 0.06
Muscle	0.07 ± 0.03	0.08 ± 0.05	0.04 ± 0.009	0.20 ± 0.09
Spleen	0.01 ± 0.01	0.05 ± 0.02	0.01 ± 0.01	0.13 ± 0.07
Small intestine	0.04 ± 0.04	0.02 ± 0.03	0.02 ± 0.02	0.08 ± 0.04
Lungs	0.12 ± 0.06	0.21 ± 0.04	0.09 ± 0.04	0.50 ± 0.14
Stomach	0.53 ± 0.13	0.49 ± 0.15	0.47 ± 0.15	0.88 ± 0.03
Tumor-to-organ ratio				
PC-3 tumor-to-liver	3.01 ± 0.25^	1.67 ± 0.56	4.00 ± 0.74^	2.12 ± 0.24
PC-3 tumor-to-kidneys	0.30 ± 0.02	0.18 ± 0.05	0.67 ± 0.13	0.39 ± 0.05
PC-3 tumor-to-muscle	28.88 ± 13.75	12.56 ± 3.75	50.68 ± 9.55	12.47 ± 0.29^

Table S5. Biodistribution of 0.75, 1.25 or 1.75 nmol [^{111}In]In-**14** (20 MBq per administered dose) in PC-3 xenografted balb/c nu/nu mice at 24 h post injection. Uptake values are expressed as percentage injected activity per gram of tissue (%IA/g) and represent the mean ± standard deviation. The asterisk shows the significance with * $p < 0.05$. $^{0.75/1.25/1.75}$ significant difference as compared with the specific applied dose for the same organ. ^ Outlier excluded from dataset based on Grubbs' test ($n = n - 1$).

**Organ/tissue**	**0.75 nmol** ($n = 4$)	**1.25 nmol** ($n = 4$)	**1.75 nmol** ($n = 4$)
Blood	0.27 ± 0.07	0.21 ± 0.05	0.19 ± 0.05
PC-3 tumor	2.74 ± 0.18	2.35 ± 0.40	2.09 ± 0.30
Prostate	0.55 ± 0.02^	0.60 ± 0.10	0.48 ± 0.01^
Pancreas	1.79 ± 0.15$^{*1.75}$	1.15 ± 0.14	1.00 ± 0.15$^{*0.75}$
Spleen	2.56 ± 1.12$^{*1.25/1.75}$	3.68 ± 0.73$^{*0.75}$	3.80 ± 0.06^,$^{*0.75}$
Liver	0.45 ± 0.23^,$^{*1.25/1.75}$	2.70 ± 1.89$^{*0.75/1.75}$	5.50 ± 1.07$^{*0.75/1.25}$
Stomach	0.60 ± 0.07	0.50 ± 0.09	0.39 ± 0.07
Small intestine	0.49 ± 0.12	0.39 ± 0.11	0.31 ± 0.05
Caecum	0.63 ± 0.12	0.47 ± 0.08	0.39 ± 0.05
Colon	0.79 ± 0.10	0.60 ± 0.08	0.45 ± 0.10
Adrenal glands	1.09 ± 0.39	1.03 ± 0.03^	0.88 ± 0.06
Kidneys	8.90 ± 0.91$^{*1.25/1.75}$	7.05 ± 1.40$^{*0.75/1.75}$	5.55 ± 0.24^,$^{*0.75/1.25}$
Lungs	0.73 ± 0.24	0.66 ± 0.18	0.56 ± 0.10
Heart	0.58 ± 0.08	0.51 ± 0.14	0.46 ± 0.08
Muscle	0.21 ± 0.05	0.18 ± 0.04	0.16 ± 0.03
Femur	0.51 ± 0.05	0.44 ± 0.08	0.39 ± 0.09
Tumor-to-organ ratio			
Tumor-to-blood	10.54 ± 2.54	11.27 ± 0.89	12.36 ± 0.31^
Tumor-to-prostate	5.04 ± 0.20^	3.94 ± 0.80	4.58 ± 0.36^
Tumor-to-liver	8.01 ± 5.46^,$^{*1.25/1.75}$	1.43 ± 1.14$^{*0.75}$	0.39 ± 0.06$^{*0.75}$
Tumor-to-kidneys	0.31 ± 0.05	0.33 ± 0.03	0.37 ± 0.07^
Tumor-to-muscle	13.87 ± 3.90	13.36 ± 1.31	13.55 ± 3.45

Table S6. Organ/tissue fluorescence after dissection of PC-3 xenografted balb/c nu/nu mice at 24 h post injection of 0.75, 1.25 or 1.75 nmol [^{111}In]In-**14** (20 MBq per administered dose). The signal is expressed as average radiant efficiency in 10^8 p/sec/cm^2/sr per µW/cm^2. The asterisk shows the significance with * $p < 0.05$. $^{0.75/1.25/1.75}$ significant difference as compared with the specific applied dose for the same organ. ^ Outlier excluded from dataset based on Grubbs' test ($n = n - 1$).

**Organ/tissue**	**0.75 nmol** ($n = 4$)	**1.25 nmol** ($n = 4$)	**1.75 nmol** ($n = 4$)
PC-3 tumor	7.30 ± 0.16^,$^{*1.25/1.75}$	10.05 ± 0.90$^{*0.75/1.75}$	12.10 ± 1.59$^{*0.75/1.25}$
Kidneys	14.73 ± 1.47$^{*1.25/1.75}$	2.48 ± 2.99$^{*0.75/1.75}$	32.15 ± 3.79$^{*0.75/1.25}$
Pancreas	2.81 ± 0.23	3.63 ± 0.40	4.15 ± 0.24
Liver	2.59 ± 0.06^,$^{*1.75}$	3.79 ± 0.50	5.27 ± 0.07$^{*0.75}$
Lungs	0.73 ± 0.20	1.01 ± 0.16	1.70 ± 0.46
Stomach	1.53 ± 0.13	1.62 ± 0.35	2.00 ± 0.26
Muscle	0.33 ±0.09	0.48 ± 0.13	0.63 ± 0.16
Spleen	0.27 ± 0.05	0.37 ± 0.07	0.58 ± 0.15
Small intestine	0.19 ± 0.04	0.27 ± 0.01^	0.39 ± 0.04
Femur	0.34 ± 0.01^	0.50 ± 0.07	0.64 ± 0.14
Tumor-to-organ ratio			
Tumor-to-liver	2.82 ± 0.13^	2.77 ± 0.02^	2.30 ± 0.32
Tumor-to-kidneys	0.52 ± 0.04^	0.41 ± 0.03	0.38 ± 0.05
Tumor-to-muscle	22.94 ± 8.28^	22.14 ± 5.79	20.38 ± 6.50

References

1. Sasmal, R.; Das Saha, N.; Pahwa, M.; Rao, S.; Joshi, D.; Inamdar, M.S.; Sheeba, V.; Agasti, S.S. Synthetic Host–Guest Assembly in Cells and Tissues: Fast, Stable, and Selective Bioorthogonal Imaging via Molecular Recognition. *Analytical Chemistry* **2018**, *90*, 11305-11314.
